# Medication-Related Osteonecrosis: Why the Jawbone?

**DOI:** 10.3390/dj11050109

**Published:** 2023-04-23

**Authors:** Sydney Kuehn, Rafaela Scariot, Mohammed Elsalanty

**Affiliations:** 1 Department of Medical Anatomical Sciences, College of Osteopathic Medicine of the Pacific, Pomona, CA 91766, USA; 2 Department of Oral and Maxillofacial Surgery, Federal Technological University of Paraná, Curitiba 80230-901, Brazil

**Keywords:** medication-related osteonecrosis of the jaw, osteoporosis medications, osteonecrosis

## Abstract

Medication-related osteonecrosis of the jaw (MRONJ) has emerged as a complication of anti-resorptive medications. Despite its low incidence rate, this problem has gained attention in recent years due to its devastating consequences and lack of preventive strategy. The fact that MRONJ incidence has been exclusive to the jawbones, despite the systemic effect of anti-resorptive medications, could be a starting point to unravel the multifactorial pathogenesis of this condition. This review aims to negotiate the question of why the jawbone is more susceptible to MRONJ than other skeletal sites. Approaching the problem from this perspective could provide new directions for the prevention of MRONJ and expand our understanding of the unique oral microenvironment.

## 1. Introduction

The pathophysiology of medication-related osteonecrosis of the jaw (MRONJ) is multifaceted, involving multiple synergistic deficiencies, including ineffective bone remodeling [1], prolonged wound healing [1], impaired immune response [2], deficient angiogenesis [3], local infection [4], and chronic inflammation [5]. Despite decades of research, there is still no clear explanation as to why MRONJ has mostly been limited to the jawbone. This review examines the possible mechanisms of localization of osteonecrosis to the jaw. Evidence from the MRONJ literature could give important clues to the unique microenvironment of the alveolar bone, with significant implications to the understanding of osteo-immune and osteo-mucosal interaction.

## 2. Medications Associated with MRONJ

Earliest reports of MRONJ recorded the pathology as a specific side effect of bisphosphonates, especially the high-dose, intravenous, and nitrogen-containing formulations. However, it gradually became evident that other anti-resorptive, as well as other classes of medications were also involved (Table 1). In this review, we will focus mainly on anti-resorptive medications since they carry the highest incidence of MRONJ.

### Anti-Resorptive Medications

A common mechanism of anti-resorptive medications is targeting the survival and/or function of bone-resorbing osteoclasts. Bisphosphonates, the most common anti-resorptive medications, are internalized by osteoclasts, where they inhibit the M-CSF pathway, preventing their differentiation, inhibiting anchorage to the cell membrane, and inducing apoptosis [1,15,16]. For a long time, osteoclast inhibition has been implicated as the main cause of MRONJ. Indeed, established MRONJ lesions demonstrate large, multinucleated osteoclasts that are inactive [17,18,19,20]. However, compensatory stimulation of osteoclastogenesis and even increased osteoclast number could be observed at different stages of MRONJ [21]. In one study, cultures of the necrotic MRONJ lesions expressed giant, hyper-nucleated osteoclasts [22]. Recent studies have suggested the direct effects of anti-resorptive medications on oral immune cells [23] and periodontal ligament stem cells [24,25]. Patients receiving IV bisphosphonates have higher bone OPG and lower TRAP and RANKL expression levels, which could signify an impaired osteoclast activation by other bone cells, such as osteoblasts and osteocytes [22,26]. Experimental and clinical studies have shown that steroid therapy, nicotine smoking, and associated systemic pathologies, such as diabetes or inflammatory joint pathology, increased the risk of MRONJ with bisphosphonate treatment [27,28].

The risk of MRONJ in patients taking bisphosphonates was significantly greater in patients receiving intravenous bisphosphonates such as Zoledronate, a difference that was attributed to their greater potency, bioavailability, and longer accumulation in bone than oral bisphosphonates [27,28,29,30]. However, newer osteoclast inhibitors, such as RANKL inhibitors (e.g., denosumab), which do not accumulate in bone, have had comparable MRONJ incidences to that of zoledronate [26,31,32,33]. Denosumab mostly dissipates from the system following a 6-month drug discontinuation [34]. However, denosumab treatment still showed a cumulative effect on the risk of MRONJ, which at 6, 12, 18, and 24 months of treatment was 4.6%, 7.7%, 17.6%, and 34.3%, respectively [35]. The isolated effect of denosumab and other anti-RANKL medications on the incidence of MRONJ is still debatable, given that many patients used them following, or in conjunction with, bisphosphonates (case below).

Overall, the benefit of using antiresorptive medications greatly outweighs the risk of MRONJ. When proper preventative measures are taken, the risks can be mediated greatly, and the patients’ quality of life can be improved.

## 3. Clinical Features and Management of MRONJ

Osteonecrosis of the jaw (ONJ) presents with a range of clinical features, ranging from pain and local inflammation to exposed necrotic bone with or without persistent infection and intra-oral fistula. The presentation typically starts with pain, swelling, pus exudation, ulcers, loose teeth, signs of infection, and altered sensation of regional nerves [4,36,37]. MRONJ is defined by the American Association of Oral and Maxillofacial Surgeons (AAOMS) as the presence of an area of exposed jawbone or necrotic bone that can be probed through an intraoral or extraoral fistula that has persisted more than eight weeks, with a history of current or previous treatment with bisphosphonates, denosumab, or anti-angiogenic therapy, and with the absence of a history of radiation therapy to the jaw or metastatic disease of the jaw [7]. It is worth mentioning that rare instances of osteonecrosis of the external auditory canal have been reported with anti-resorptive treatment, which will not be discussed here [38,39,40].

**Figure 1:**
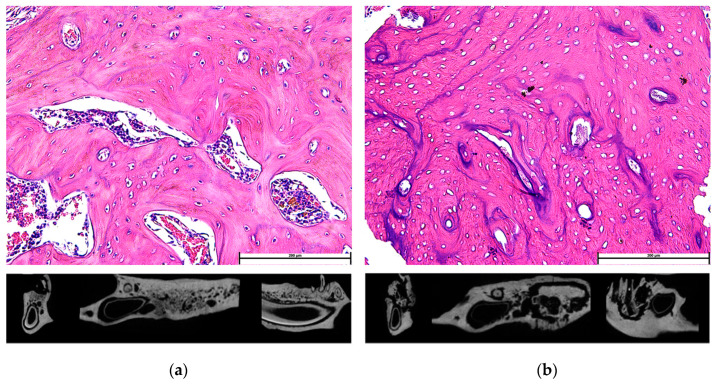
The standard method in quantifying MRONJ in experimental animals: (**a**) histology image of normal alveolar bone tissue, with the vast majority of osteocyte lacunae occupied with viable osteocytes (hence, the normal-looking nuclei); (**b**) alveolar bone in zoledronate-treated rat showing reduced viability, evident by the large percentage of empty osteocyte lacunae. Some studies confirmed bone sequestration with microCT scans (right-side microCT confirmed dense bone segment that is separated from the remainder of the alveolar bone in all three dimensions (coronal, cross, and sagittal, from left to right), as opposed to normal continuity of bone in the control jaw microCT scan on the left).

In experimental animals, the standard method to quantify MRONJ has been histomorphometry (Figure 1). In human patients, the stage of MRONJ is based on clinical presentation. Stage 0 is defined as no clinical evidence of necrotic bone, but the patient presents with non-specific symptoms or radiographic findings. The treatment strategy, in this case, involves pain medication and possibly antibiotics. Stage 3 is the most severe where the patient presents with infection and maxillary sinus involvement. The prognosis for stage 3 is very poor, and often surgical intervention is the main option [7]. Table 2 summarizes the treatment recommendations by different professional organizations.

Ideally, the management of MRONJ should focus on prevention. Effective preventive measures included preemptive dental treatment and extraction, maintaining good oral hygiene, appropriate diet, reducing alcohol intake, and smoking cessation [29,33,36,46,47,48]. Regular dental care every 3 to 6 months is important to monitor and detect early symptoms and signs [46]. It is advised that patients be referred to dental preventative care for screening and treatment before initiating any of the known medications associated with MRONJ [4]. Similarly, the status of patient dentition, existing intraosseous implants, and fit of dentures, if applicable, are important factors to consider. It is most important to identify local dental infections, especially marginal and apical periodontitis, as these confer the highest associated risk for MRONJ development [7,47]. The diagnosis and resolution of these infections should be preceding, ideally 14–21 days before, the start of antiresorptive treatment [32,46,49]. Dental extraction, when necessary, should be performed with the least trauma or be performed in a surgical setting. Antibiotic prophylaxis has been used with little evidence of efficacy in MRONJ prevention [7,32].

In established MRONJ, palliative care aims to control infection and pain, as well as excision of bone necrosis and pain [4,36,47,48]. Complete removal of necrotic bone, smoothing of sharp bone, and pristine wound closure, accompanied by antibiotic treatment, is considered the most beneficial approach to achieve MRONJ resolution [36]. Recent studies have suggested that this type of surgical intervention is useful at any stage of MRONJ development, and not only in the most severe case [7,50]. Conservative therapy not only has a low success rate, but it also requires a longer time to cure than surgery. It has been shown that the 1-year cumulative cure rate is 64.7% for surgical therapies and only 18.2% for conservative treatment [34].

Recent experimental and clinical studies tested the efficacy of adjunctive treatments to prevent and/or manage MRONJ, such as autologous leukocyte-rich and platelet-rich fibrin [51,52], bone morphogenetic protein-2 [53], photomodulation therapy [54,55], ozone therapy [56], and piezoelectric stimulation [57,58]. These studies and others presented a promising potential for these treatments. However, they have not alleviated the necessity of surgical excision, which remains the standard management strategy for established MRONJ [55].

### Drug Holiday

As early as the first reports of MRONJ, legitimate concerns were raised concerning the safety of dental procedures in patients on long-term anti-resorptive medications, especially the ones that carried the highest risk, such as intravenous bisphosphonates. A logical strategy to manage these patients has been to stop anti-resorptive treatment for a certain period prior to performing any invasive dental procedures. The duration of this “drug holiday” ranged from two months to two years [4]. Based on the information summarized in the preceding sections, there are multiple concerns with this premise.

As mentioned earlier, bisphosphonate molecules accumulate in the bone matrix, with the phosphonate group acting as a hook. The hydroxyl groups in drugs such as zoledronate may increase binding affinity and therapeutic potency [59]. Research shows that these matrix-bound bisphosphonate molecules have higher concentrations at specific bone sites for years, including the jawbone and proximal tibia, among others, and that they are biologically accessible and active [59]. Therefore, while a drug holiday may be useful to eliminate the systemic effects of bisphosphonates, it is unlikely to influence the activity of those locally deposited bisphosphonate molecules. In addition, while some studies showed an improvement in MRONJ incidence in experimental animals [60], clinical data do not support the efficacy of drug holidays in reducing the risk of MRONJ in bisphosphonate-treated patients, while depriving the patient of the therapeutic effects of these medications [34,61,62].

On the other hand, discontinuation of denosumab before dental procedures was associated with a drastic rebound in the rate of osteoporotic fractures [35]. Evidence showed a steep rise in bone remodeling, not only eliminating the treatment effects but further compromising the mechanical integrity of bone [63]. Discontinuation of denosumab treatment was associated with a 3–5-fold increase in the risk of a major osteoporotic fracture [64]. The markers of bone breakdown and osteoclast activity increased in these patients [65].

Again, studies that separately examined both bisphosphonates and denosumab found that drug holiday had no effect in either group on the incidence of MRONJ [34,66]. Therefore, weighing the risks involved in discontinuing treatment against the lack of evidence for a reduction in MRONJ, it has been suggested that necessary dental procedures could be performed without discontinuing the use of anti-resorptive therapy [34]. The debate around this point still continues.

## 4. MRONJ following Implant Placement—A Case Report

**Figure 2:**
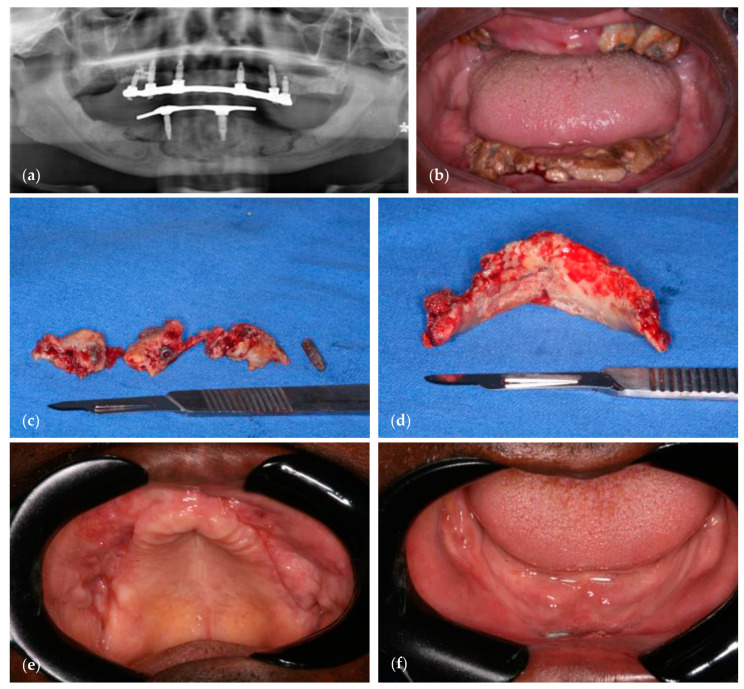
A 59-year-old male patient with multiple myeloma and a history of zoledronate treatment for 3 years, followed by Denosumab treatment for 3 years. Treatment stopped a year before the patient had multiple implants in the maxilla, and then in the mandible 4 months later, for a full denture. Shortly after, massive osteonecrosis occurred in both the mandible and maxilla. (**a**) Panoramic reconstruction of cone-beam computerized tomography of the mandible and maxilla, demonstrating sequestration of alveolar bone in both jaws; (**b**) intraoral photograph showing exposed necrotic bone; (**c**,**d**) sequestered bone removed from the maxilla and mandible, respectively; (**e**,**f**) intraoral photographs one month after surgical excision and primary closure of the maxilla and mandible, respectively, showing complete mucosal healing.

A 59-year-old male patient had a history of multiple myeloma, for which zoledronate treatment was administered for 3 years. Zoledronate was then replaced by Denosumab treatment for another 3 years. Treatment stopped a year before the patient had multiple implants in the maxilla, and then in the mandible 4 months later, for a full denture. Shortly after the implant procedure, massive osteonecrosis developed in both the mandible and maxilla. Surgical treatment involved the excision of necrotic bone, followed by the smoothening of bone edges, and primary mucosal closure. Amoxicillin and Metronidazole were administered following the surgery. Figure 2 presents the details of the case.

In this case, alveolar bone was clearly compromised, despite the following facts: (1) the patient was malignancy-free; (2) it had been four years since Zoledronate stopped and one year since Denosumab stopped; (3) there was no preexisting infection or inflammation prior to the procedure. This case is one of many that demonstrate clearly that alveolar bone itself could be intrinsically compromised due to anti-resorptive medication, a problem that seems to be unique to this skeletal site.

This result is in concordance with other reports that highlight the risk of implant placement in patients with a history of anti-resorptive medications [67,68]. Other reports, however, suggested that complications were limited to manageable peri-implantitis [69,70]. Further large-scale studies are needed to determine predictive parameters for the feasibility and safety of implant placement in patients with a history of antiresorptive medication treatment.

## 5. Potential Mechanisms for Localization of Osteonecrosis

Based on the current knowledge of MRONJ summarized above, the goal should be to develop a strategy to prevent MRONJ while preserving the critically important therapeutic effects of anti-resorptive medications. One clue to unlocking this strategy would be to pursue the localizing factors that make the jawbone uniquely vulnerable to osteonecrosis with long-term anti-resorptive therapy.

In patients receiving anti-resorptive medications, osteonecrosis was never reported in long bones. In Zoledronate-treated rats, traumatic bone exposure heals normally in areas where localized accumulation of the drug was comparable to that of the jawbone [71]. On the other hand, Zoledronate treatment was associated with anabolic changes in cortical and trabecular areas in the long bone, indicating that bone formation and remodeling remain active at normal levels in these areas [2]. While levels of Wnt-3a and RANKL were significantly decreased in the jawbones of Zoledronate-treated animals, iliac and tibial bones exhibited significantly increased levels [2].

Many factors have been explored as possible localizing factors, including dental trauma, especially surgical extraction, periodontitis, impaired gingival healing, changes in oral bacteria biofilm profile, and impaired innate immune response specific to the oral cavity [19,28,33,36,37,72,73,74,75,76,77,78].

### 5.1. Dental Trauma

The incidence of MRONJ was highest with invasive dental trauma, such as tooth extraction, or minor oral or periodontal surgery (51–82%) [72,75,76,77,79,80,81,82,83]. While dental extraction seemed to top the list of risk factors for MRONJ [84], it could be argued that dental extraction was almost always preceded by chronic periodontitis, infection, or severe caries [80]. Indeed, some clinical data suggested that the rate of MRONJ actually decreased if the infected tooth was extracted, even without stopping anti-resorptive therapy [35,85]. However, many studies found that the risk of MRONJ with dental trauma was very high in association with local infection, abscesses, and poor oral hygiene [27,33,37,72,73,74,75,76,78,83,86,87,88,89]. Recent studies have shown that not only post-treatment implant placement but also the pre-existence of osseointegrated implants prior to therapy can be associated with MRONJ [90,91]. Posterior implants seemed to carry the highest risk [90,92], and the mandible was more susceptible than the maxilla [93]. Overall, as in the presented case report above, it seems that implants, whether new or preexisting, increase the risk of MRONJ. In patients undergoing tooth extraction after long-term anti-resorptive treatment, the risk of MRONJ varied between 1 and 5% [80]. The likelihood of MRONJ seemed to increase with subsequent extractions and with a longer duration of anti-resorptive use [33,79,82]. It is also worth noting that spontaneous MRONJ (without a history of any of the abovementioned risk factors) still occurred in as many as 35.1% of patients [5].

### 5.2. Chronic Inflammation in the Oral Cavity

In experimental animals, ligature-induced periodontitis and tooth extraction were widely used, separately or combined, to induce osteonecrosis in rats or mice treated with anti-resorptive medications. In these studies, dental extraction alone induced MRONJ lesions of various severity, while pre-existing pathologic inflammation increased the incidence and severity of MRONJ development following extraction with both Zol and Anti-RANKL treated animals [76,87,88,94,95]. It is now widely accepted that poor oral hygiene and chronic bacterial infection of the bone surface contributed to disease severity [27,33,80,96]. Since dental extraction is a standard treatment of chronic periodontitis, there is a confounding relationship between the two factors in MRONJ [27,80,89,97]. Chronic periodontal inflammation is associated with the suppression of bone healing through multiple mechanisms [41]. Evidence suggests that the jawbone may be more susceptible to chronic infection compared to other bones in the body due to its exposure to exponentially more bacteria [41,97]. In addition, MRONJ lesions invariably originated from the alveolar process of the jaw, which is frequently affected by odontogenic infections through the root tips or infected periodontal pockets [27].

The strong role of local inflammation in MRONJ has been supported by the presence of inflammatory cytokines at the site of necrosis [98]. In numerous studies, mice with experimentally induced rheumatoid arthritis demonstrated more severe MRONJ with larger areas of exposed bone and more severe necrosis [99]. The transplantation of peripheral blood mononuclear cells with anti-inflammatory properties reduced MRONJ prevalence and was associated with improved healing, decreased inflammatory cell infiltration, and enhanced vascularity [80]. On the other hand, anti-resorptive medications, such as bisphosphonates, were shown to enhance pro-inflammatory cytokine production and release, and therefore, sustain an inflammatory state [98,100].

### 5.3. Oral Mucosal Barrier

The oral mucosal barrier involves complex immune system components that constantly recognize and eliminate harmful exogenous organisms while maintaining commensals [101]. It has been hypothesized that bisphosphonates disrupt the local immune response through the inhibition of dendritic cell function [23,102]. One study found that bisphosphonate treatment led to increased oral bacterial load, accompanied by a significant decrease in antigen-presenting cells and decreased dendritic cell differentiation and activity [23]. It also found that dendritic cell-deficient mice contributed to an increased rate of MRONJ development [23]. Therefore, bisphosphonate-related disruption of the innate oral immune system can lead to colonization of the mucosa with pathogenic bacteria and perpetuate necrosis following dental trauma. Furthermore, bisphosphonates may be internalized by macrophages, impacting their phagocytic ability [20,103]. Macrophages treated with zoledronic acid have shown decreased protein expression and higher apoptotic rates which are furthered by LPS stimulation from gram-negative bacteria that have been implicated in MRONJ [103,104]. On the other hand, bisphosphonates enhance pro-inflammatory cytokine production and release, and therefore, sustain an inflammatory state [100]. Recent reports and ongoing studies demonstrated that T-cell dysregulation may play an important role in the pathogenesis of MRONJ [105].

### 5.4. Oral Bacteria

Interestingly, there is evidence that the depletion of indigenous oral microbes and the resulting dysbiosis could increase the risk of osteonecrosis induced by bisphosphonates following tooth extraction [33,76,106]. In other words, oral bacterial flora could play a role in the homeostatic mechanisms of the alveolar bone that are protective against osteonecrosis. Thus, a balance must be maintained between the management of infection and the preservation of the local oral flora [2,106]. This is especially relevant since the use of prophylactic antibiotics is a very common practice with invasive dental procedures. On the other hand, a sequestered bone from MRONJ lesions has consistently shown microbial biofilm formation, with Actinomycetes detected in 70% of samples [107,108]. Several studies have linked MRONJ to Actinomyces, especially Aggregatibacter Actinomycetemcomitans, which are common in patients with periodontitis, again linking MRONJ to the underlying prevalence of these bacteria [27,96,102]. It has been hypothesized that the mechanism of trauma-induced MRONJ simply includes creating access for pre-existing pathogenic bacteria [109], which is why MRONJ almost always started at the alveolar process of the jawbone. Gram-negative bacteria in particular are thought to produce toxic products, such as lipopolysaccharides that directly induce osteoclast differentiation and activity [107]. Other bacteria associated with MRONJ development included Prevotella and Fusobacterium [109].

Despite the concern about dysbiosis resulting from prophylactic antibiotics, mentioned above, studies have shown that the use of antibiotics could indeed help after MRONJ has already started, along with surgical debridement [110]. Therefore, antibiotics have become standard in patients with ongoing MRONJ.

As detailed in previous sections, bisphosphonate molecules accumulate in the bone, where they remain for many years after stopping treatment [26,79,111]. These deposits have been shown to increase the adhesion of different bacterial species and promote biofilm formation [27]. Experimental evidence showed that osteonecrosis could be induced following invasive trauma in long bones of zoledronate-treated animals, but only in the presence of pre-existing Actinomyces infection [71,76]. Our studies and others have matrix-bound bisphosphonate molecules that are biologically accessible and active in vivo [59,111,112]. Therefore, deposited bisphosphonates, which have been shown to preferentially adhere to alveolar bone more than basal bone [112], maybe an attractive surface for bacterial colonization and formation of pathologic biofilm when bisphosphonate loaded surfaces are exposed, such as following invasive dental procedures [59].

### 5.5. Other Forms of Osteonecrosis

Finally, other forms of osteonecrosis have been described at different skeletal sites. For example, osteonecrosis occurs in the femoral head and is described as avascular necrosis, and it is not associated with anti-resorptive medications [80]. However, even though vascular impairment has been described in MRONJ, it seems to be only one aspect of a multifactorial process that starts primarily in the bone tissue itself. Indeed, MRONJ lesions show a decrease in vascularity during bone healing, which seems to be another effect of bisphosphonates [80,113]. When osteonecrosis occurs in the femur, it is not accompanied by bone exposure or infection. A similar difference exists between MRONJ and osteoradionecrosis. The contrast in pathogenesis between MRONJ, avascular necrosis, osteoradionecrosis, and osteomyelitis-related osteonecrosis is beyond the scope of the current review.

## 6. Conclusions

The pathophysiology underlying MRONJ seems to involve a myriad of systemic and local factors. At a certain point, the alveolar bone becomes intrinsically compromised. Triggering factors, such as trauma and periodontal infection, could induce osteonecrosis in already compromised bone. Beyond the systemic effects of anti-resorptive medications, alveolar bone is uniquely susceptible, likely due to the combination of its proximity to oral microbiota and interaction with the oral immune system, both of which are dysregulated by the medications. In addition, the chronicity of inflammation in dental tissues and the unique dental trauma present a particular challenge for the already compromised homeostatic mechanisms of the underlying bone. Specific to intravenous bisphosphonates, the locally deposited bisphosphonate molecules continue to compromise alveolar bone for years after stopping treatment. This summary review was limited to MRONJ and did not involve other forms of osteonecrosis. Ongoing studies continue to unravel the role of the oral immune response in alveolar bone homeostasis and how it might contribute to the induction of MRONJ.

## Figures and Tables

**Table 1:** Medications associated with the development of MRONJ as of 2023 [6,7,8,9,10,11,12,13,14].

Class	Drug	Use
Bisphosphonates	Etidronate	Skeletal dysplasia, osteoporosis, hypercalcemia, bone metastasis
	Clodronate	
	Alendronate	
	Risedronate	
	Tiludronate	
	Pamidronate	
	Ibandronate	
	Zoledronate	
RANKL Antibody	Denosumab	Osteoporosis, pathologic fracture, hypercalcemia, bone metastasis
Anti-VEGF	Bevacizumab	Anti-cancer, diabetic retinopathy
	Aflibercept	
	Pazopanib	
	Cabozantinib	
Anti-TKIs	Sunitinib	Anti-cancer, idiopathic pulmonary fibrosis
	Axitinib	
	Dasatinib	
	Imatinib	
	Erlotinib	
	Sorafenib	
Anti-TNF	Infliximab	Autoimmune conditions
	Adalimumab	
Anti-CD20	Rituximab	B-cell proliferative disorders, non-Hodgkin’s lymphoma, and chronic lymphocytic leukemia
Immunomodulators	Methotrexate	Anti-cancer, psoriasis, inflammatory bowel disease, rheumatoid arthritis
mTOR inhibitors	Temsirolimus	Transplant rejection prevention, advanced breast, kidney, and leukemia
	Everolimus	

**Table 2:** Organizational guidelines surrounding the management, prevention, and diagnosis of MRONJ in patients taking antiresorptive or anti-angiogenic medications [38,39,40,41,42,43,44,45].

Organization	Medications	Management	Prevention	Diagnosis
American Association of Oral and Maxillofacial Surgeons	Antiresorptive and anti-angiogenics	Removal of necrotic bone, extraction of symptomatic teeth	Delay of medication until after dental work, dental hygiene	Staging based on exposed necrotic bone and fistula formation with infection
International Osteoporosis Foundation	Antiresorptive and anti-angiogenics	Tooth extraction, teriparatide, control inflammation	Encouraging high bone mass, dental hygiene, antibiotic prophylaxis	Staging based on the level of bone exposure
Italian Allied Committee on ONJ	Antiresorptive and anti-angiogenics	Antibiotic prophylaxis and inflammation control	Dental hygiene, extraction of teeth before medication	Radiology to determine the extent of skeletal disease
Korean Association of Oral and Maxillofacial Surgeons	Antiresorptive and anti-angiogenics	Control inflammation, pain alleviation, removal of necrotic tissue, teriparatide	Drug holiday, dental hygiene	Staging based on exposed necrotic bone and fistula formation with infection
Multinational Association of Supportive Care in Cancer	Antiresorptive and anti-angiogenics	Antibiotics, removal of necrotic bone, aggressive surgical intervention	Elective dental surgery, drug holiday, hygiene	Staging based on exposed necrotic bone and fistula formation with infection

## Data Availability

No new data were created.
